# Biochemical Changes in Leaves of *Vitis vinifera* cv. Sangiovese Infected by Bois Noir Phytoplasma

**DOI:** 10.3390/pathogens9040269

**Published:** 2020-04-07

**Authors:** Carmine Negro, Erika Sabella, Francesca Nicolì, Roberto Pierro, Alberto Materazzi, Alessandra Panattoni, Alessio Aprile, Eliana Nutricati, Marzia Vergine, Antonio Miceli, Luigi De Bellis, Andrea Luvisi

**Affiliations:** 1 Department of Biological and Environmental Sciences and Technologies, University of Salento, 73100 Lecce, Italy; carmine.negro@unisalento.it (C.N.); erika.sabella@unisalento.it (E.S.); alessio.aprile@unisalento.it (A.A.); eliana.nutricati@unisalento.it (E.N.); marzia.vergine@unisalento.it (M.V.); antonio.miceli@unisalento.it (A.M.); luigi.debellis@unisalento.it (L.D.B.); andrea.luvisi@unisalento.it (A.L.); 2 Department of Agriculture, Food and Environment (DAFE), University of Pisa, 56100 Pisa, Italy; rob.pierro@outlook.it (R.P.); alberto.materazzi@unipi.it (A.M.); alessandra.panattoni@unipi.it (A.P.)

**Keywords:** Bois noir, grapevine cultivar (cv.) Sangiovese, plant-phytoplasma interaction, phenylpropanoid compounds

## Abstract

Bois noir is a disease associated with the presence of phytoplasma ‘*Candidatus* Phytoplasma solani’ belonging to the Stolbur group (subgroup 16SrXII-A), which has a heavy economic impact on grapevines. This study focused on the changes induced by phytoplasma in terms of the profile and amount of secondary metabolites synthesized in the phenylpropanoid pathway in leaves of *Vitis vinifera* L. red-berried cultivar Sangiovese. Metabolic alterations were assessed according to the disease progression through measurements of soluble sugars, chlorophyll, and phenolic compounds produced by plant hosts, in response to disease on symptomatic and asymptomatic Bois noir-positive plants. Significant differences were revealed in the amount of soluble sugars, chlorophyll, and accumulation/reduction of some compounds synthesized in the phenylpropanoid pathway of Bois noir-positive and negative grapevine leaves. Our results showed a marked increase in phenolic and flavonoid production and a parallel decrease in lignin content in Bois noir-positive compared to negative leaves. Interestingly, some parameters (chlorophyll *a*, soluble sugars, total phenolic or flavonoids content, proanthocyanidins, quercetin) differed between Bois noir-positive and negative leaves regardless of symptoms, indicating measurable biochemical changes in asymptomatic leaves. Our grapevine cultivar Sangiovese results highlighted an extensive modulation of the phenylpropanoid biosynthetic pathway as a defense mechanism activated by the host plant in response to Bois noir disease.

## 1. Introduction

Phytoplasmas are obligate plant-pathogenic organisms of the Mollicutes class, which infect many crops and hundreds of plant genera cultivated worldwide; thus, causing heavy economic losses [1,2]. Phytoplasmas have become an increasingly serious threat to many perennial plants, such as fruits, shrubs, woods, ornamental trees and, in particular, grapevine in Europe. In Italy, which is the second-largest wine producer in Europe, phytoplasmas are a serious limiting factor for grape production, particularly in traditional grapevine-growing regions [3]. 

One of the most important diseases in the main viticultural areas of Italy is “Bois noir” (BN), caused by the phytoplasma ‘*Candidatus* Phytoplasma solani’ of the Stolbur group (subgroup 16SrXII-A) [4]. This strain is transmitted from plant-to-plant above all by *Hyalesthes obsoletus* Signoret (Homoptera: Cixiidae), a polyphagous leafhopper, which has various hosts that act as phytoplasma reservoirs [5].

The symptoms on infected plants depend on the variety, but generally, different visual symptoms are exhibited, such as stunting, abnormal lignification of canes, short internodes, flower abortion, downward curling, and yellowing and/or reddening leaves, resulting in severe yield reduction and even plant death [6]. 

A number of mechanisms by which phytoplasmas induce disease symptoms have been proposed: through the production of phytoplasma-secreted proteins (effectors), which play an important role in host-pathogen interactions and pathogenicity eliciting the disease symptoms [7]. The latter include yellowing or reddening leaves, in relation to white- or red-berried grape varieties respectively, induced by phytoplasma are associated with an alteration in the use of sugars in the phloem [8,9]. In fact, several studies have reported evidence of extensive modification in the synthesis and transport of soluble carbohydrates and starch, a decrease in photosynthetic activity, the breakdown of chlorophyll, carotenoids, and their biosynthesis inhibition in many phytoplasma-infected plants, including in Vitis spp. [8,10,11,12,13]. In BN-infected grapevine of white-berried cv. Chardonnay, symptoms have been related to reduced photosynthetic activity and the anomalous accumulation of carbohydrates [8], and down-regulated photosynthetic genes and up-regulated defense genes [11].

These metabolic alterations, such as the biosynthesis inhibition of chlorophyll, reduction in photosynthetic activity and the accumulation of soluble carbohydrates (sucrose) and starch in the leaves of the infected plant [9,14] can lead to a modification of secondary metabolism, especially of phenylpropanoid biosynthesis [15]. In fact, in parallel to the alteration in primary carbohydrate metabolism, metabolites of the secondary metabolism pathways are induced, such as derivates of shikimic acid and genes involved in direct defense responses. Some studies have reported the accumulation or reduction of secondary metabolites, such as phenolics and flavonoids, and in particular of specific compounds (resveratrol glucoside, anthocyanin, and lignin) in relation to phytoplasma infection [16,17,18,19]. The roles of phenolics and flavonoids in plant-pathogen interactions have been the subject of numerous studies that have described how stress-induced compounds (such as signaling of defense responses, protection against UV light damage, increase in the bioavailability of recalcitrant nutrients) play an important role in resistance to pathogen attack by acting as a quencher of the host plant’s defense reactions [18,20,21]. However, most studies focus on white-berried grape cultivars, which have different symptoms (yellow and chlorotic leaves) from red-berried grape cultivars (purple-reddish leaves).

The aim of this study was to investigate the effect of BN disease on the primary and secondary metabolism parameters in leaves of a yet untested red-berried grape cv. Sangiovese, which is one of the most widespread Italian cultivars whose susceptibility to BN has been reported [22,23]. Specifically, the sugar accumulation, photosynthetic pigments and the compounds of phenylpropanoid pathways, such as phenolic compounds, flavonoids, proanthocyanidins, anthocyanins, and lignin, and their respective amounts were evaluated in BN-positive and BN-negative leaves, in two periods, according to symptom appearance (asymptomatic or symptomatic).

## 2. Results

### 2.1. Plant Symptoms

**Figure 1:**
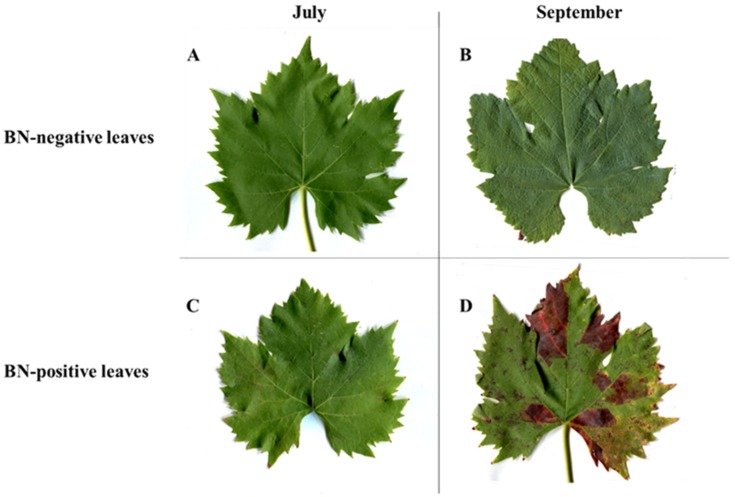
Leaves of grapevine cv. Sangiovese BN-negative and BN-positive: (**A**) BN-negative leaves collected in July; (**B**) BN-negative leaves collected in September; (**C**) BN-positive leaves collected in July (sampled from plants with symptom severity class 0 (plants with no symptoms); (**D**) BN-positive leaves collected in September (symptoms of disease on leaves were clearly visible and were sampled from plants with symptom severity class 3 (more than three shoots with reddening leaf and berry shrivel)). BN = Bois noir phytoplasma.

Figure 1 shows BN-positive leaves with typical symptoms, such as different degrees of severity of discolored veins and laminas, and interveinal reddening. The figure also shows the BN-negative leaves of grapevine cv. Sangiovese. In July, BN-positive samples were collected from plants showing symptoms of severity class 0 (= plants with no symptoms, Figure 1C), while in September, the same plants were classified as belonging to severity class 3 (= more than three shoots with symptoms of reddening leaf, Figure 1D). Thus, BN-positive samples collected in July were considered as asymptomatic, while those collected in September were considered as symptomatic.

### 2.2. Pigments and Soluble Sugars

The amount of chlorophyll *a* measured in BN-negative leaves was higher than in BN-positive leaves in two different key-stages. This, thus, showed the better photosynthesis activity of BN-negative leaves and a reduction in its amount in the BN-positive leaves from −15% to −44% from July to September (Table 1). On the other hand, the measured quantity of chlorophyll *b*, total chlorophylls (*a*+*b*), and carotenoids (Cars), despite being lower in the BN-positive leaves compared to BN-negative leaves in July, was considerably less in September, when the symptoms of phytoplasma disease were clearly visible on the leaves. The Chl *a*/*b* ratio decreased in BN-positive leaves, from −14% in July to −19% in September (Table 1), while the Car/Chls ratio was unaffected and no differences between BN-negative and BN-positive leaves were recorded (Table 1).

The soluble sugars detected in BN-positive leaves were higher than the BN-negative leaves in two different key-stages. In particular, in the BN-positive leaves, the soluble sugar content was over +68% in BN-positive leaves sampled in July, and + 31% in BN-positive leaves sampled in September, as reported in Table 1. 

### 2.3. Total Phenolic Content (TPC), Total Flavonoid Content (TFC), and Proanthocyanidins (PAs)

**Figure 2:**
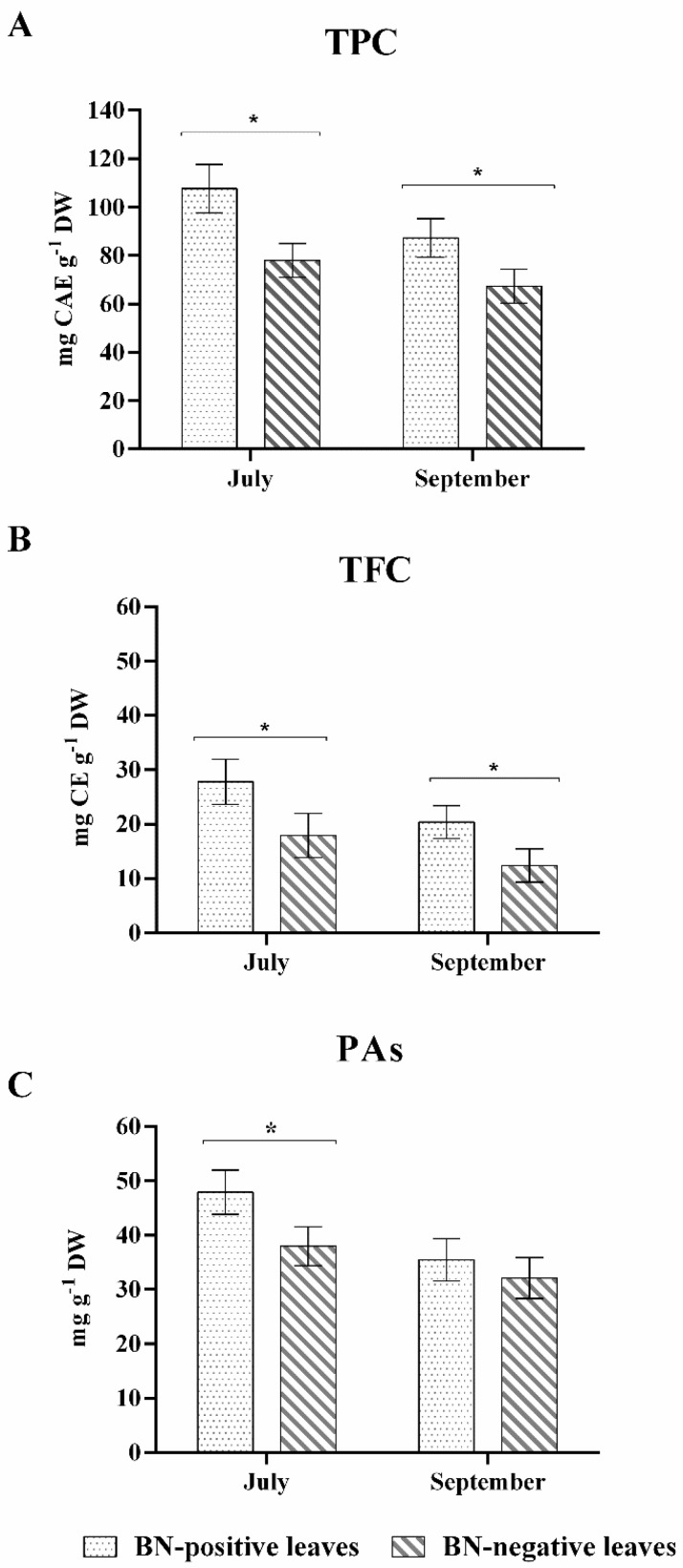
(**A**) total phenolic content (TPC) expressed as mg of caffeic acid equivalent (CAE)·g^−1^ dry weight (DW) (**B**) total flavonoid content (TFC) expressed as mg of catechin equivalent (CE)·g^−1^ DW and (**C**) proanthocyanidins (PAs) (mg·g^−1^ DW) in BN-positive and BN-negative leaves collected in July and September. The statistical analysis between BN-positive and BN-negative leaves was carried out using a multiple *t*-test (FDR = 5%) and significant differences at *p* < 0.05 are marked by an asterisk. Values are reported as means and standard deviations of five harvested samples (n = 5 BN-positive and BN-negative plants, respectively), each measured in three technical replicates. BN = Bois noir phytoplasma.

Statistically significant differences in TPC and TFC leaf extracts were observed between BN-positive and BN-negative leaves at the key stages analyzed (Figure 2). TPCs measured in BN-positive samples collected in July and September were +38% and +29% higher compared to BN-negative leaves, respectively. Similarly, TFCs measured in two key-stages were +55% and +65% higher in BN-positive leaves compared to BN-negative leaves. These results indicated higher amounts of both total phenolics and flavonoids in BN-positive leaves regardless of the presence of disease symptoms on leaves. 

On the other hand, the differences between the BN-negative and BN-positive leaves in PA amount was statistically significant only in July with higher values in the BN-positive leaves (+26%) despite the samples not clearly showing symptoms of BN disease. The BN-positive leaves sampled in September, with clear symptoms of phytoplasma disease, did not show differences in the amount of PAs compared to the BN-negative leaves sampled at the same stage (Figure 2).

### 2.4. Quali-Quantitative Analysis of Phenolic Compounds and Anthocyanins in Leaf Extracts

Table 2 shows the phenolic compounds and anthocyanins extracted from the BN-positive and BN-negative leaves of grapevine cv. Sangiovese and identified by negative and positive ionization mode, using High-Performance Liquid Chromatography coupled to Electrospray Ionization Time-of-Flight Mass Spectrometry (HPLC ESI/MS-TOF). The qualitative analysis of all the extracts identified 42 compounds, which are listed in Table 2 with the respective retention times, experimental and calculated *m*/*z*, molecular formula, errors, score, and literature references.

The qualitative analysis of the cv. Sangiovese BN-positive and BN-negative leaf extracts did not show significant differences, indicating that there was no evident qualitative variation between the phenolic and anthocyanin profiles identified in our study. In the extracts, 42 compounds were detected, 36 of which were annotated or identified. However, only compounds that have differences lower than 5 ppm are considered properly annotated (Table 2).

The compounds analyzed, listed in Table 2, belonged to seven different chemical classes, dihydroxybenzoic acids: protocatechuic acid 3-glucoside (n. **1**), protocatechuic acid (n. **4**), and protocatechuic acid-glucoside (n. **5**); hydroxycinnamic acids: cafataric acid isomer 1-2 (n. **2, 3**), rosmarinic acid (n. **6**), caffeic acid glucoside (n. **7**), tartaric (n. **8**), and coumaric acid (n. **9**); flavan-3-ols: catechin (n. **10**), epicatechin (n. **15**), and caffeic acid and catechin condensation product (n. **37**); flavonols: isorhamnetin 3-glucuronide isomer 1-2 (n. **11, 35**), myricetin 3-*O*-glucuronide isomer 1-3 (n. **16, 22, 23**), quercetin-pentoside (n. **19**), myricetin 3-glucoside (n. **24**), quercetin-glucoside isomer 1-2 (n. **26, 28**), quercetin 3-glucuronide (n. **27**), kaempferol 3-*O*-glucoside (n. **29**), kaempferol-rutinoside (n. **31**), kaempferol 3-*O*-glucuronide (n. **32**), quercetin 3-*O*-rhamnoside (n. **33**), quercetin (n. **38**) and syringetin-3-*O*-galactoside (n. **39**); sugars: hexose derivate isomer 1-3 (n. **17**, **18**, **20**); stilbenoids, only 5.56% of total compounds detected (viniferin (n. **12**) and resveratrol glucoside (n. **36**)) and anthocyanidins: delphinidin 3-glucoside (n. **40**), cyanidin 3-glucoside (n. **41**), and peonidin 3-glucoside (n. **42**). 

Quantitative analyses were performed on the most representative secondary metabolites commonly associated with phytoplasma disease in grapevines.

**Figure 3:**
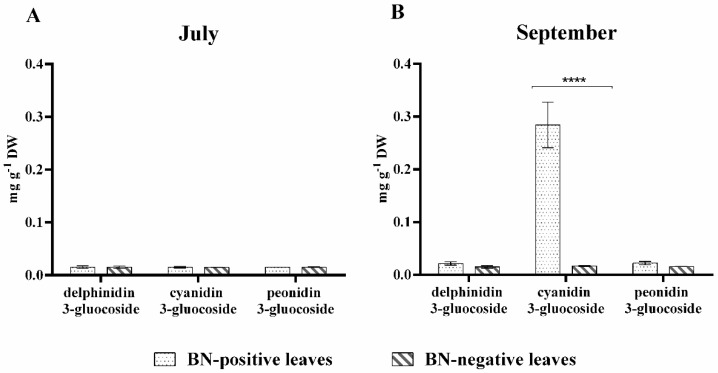
Amount of anthocyanidins (mg·g^−1^ dry weight (DW)) in BN-positive and BN-negative leaves: (**A**) leaves collected in July (symptoms of disease on leaves were not yet visible) and (**B**) leaves collected in September (symptoms of disease on leaves were clearly visible). The statistical analysis between BN-positive and BN-negative leaves was carried out using multiple t-test (FDR = 5%) and significant differences at *p* < 0.0001 are marked by four asterisks. Values are reported as means and standard deviation of five harvested samples (n = 5 BN-positive and BN-negative plants, respectively), each measured in three technical replicates. BN = Bois noir phytoplasma.

*Anthocyanins*. The three anthocyanins, delphinidin 3-glucoside (n. **40**), cyanidin 3-glucoside (n. **41**), and peonidin 3-glucoside (n. **42**), identified in the leaf extracts (Table 2), were quantified and the respective amounts are shown in Figure 3. 

As shown in Figure 3, all three anthocyanins were detected only in traces in the BN-negative leaves sampled both in July and in September. On the other hand, in BN-positive leaves, in July the anthocyanins were present only in traces, while in September, there was a considerable accumulation only of cyanidin 3-glucoside (n. **41**). In fact, when in September, on the BN-positive leaves the symptoms of disease were clearly visible (Figure 1), the amount of cyanidin 3-glucoside (n. **41**) was more than 10-fold higher than the BN-negative leaves, where no chlorotic spots and reddish-purple coloration of leaves were observed. In September, however, the leaves collected from the same plants did not show an accumulation or statistically positive variation in delphinidin 3-glucoside (n. **40**) and peonidin 3-glucoside (n. **42**), the other two anthocyanins quantified, compared to BN-negative leaves and/or compared to the previous sampling performed in July (Figure 3).

**Figure 4:**
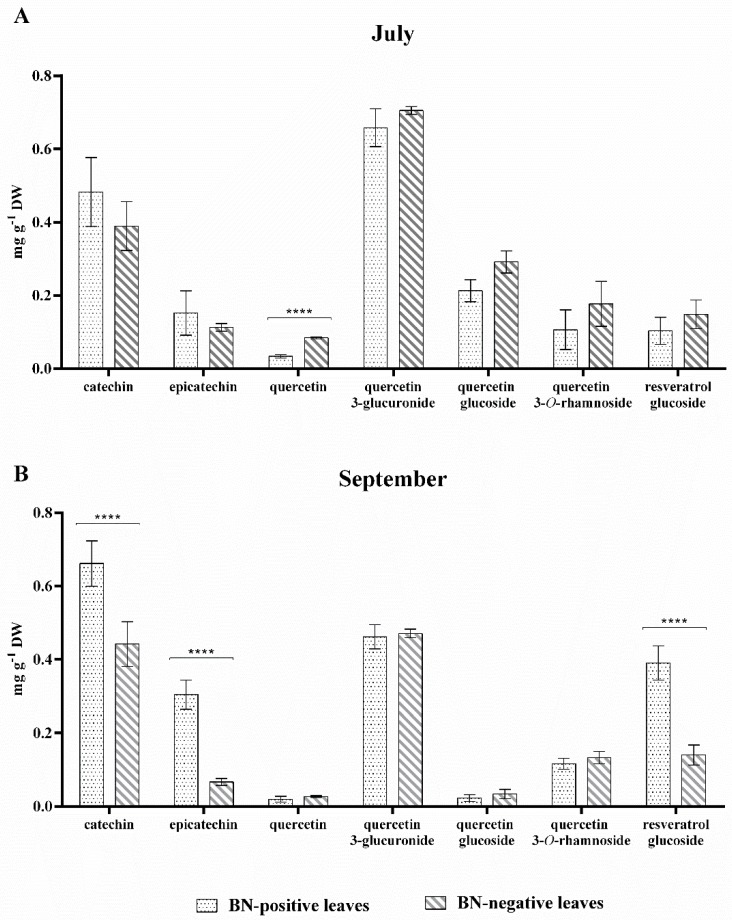
The content of flavan-3-ols (catechin and epicatechin), flavonols (quercetin, quercetin 3-glucuronide, quercetin-glucoside, and quercetin 3-*O*-rhamnoside), and stilbenoid (resveratrol glucoside) in BN-positive and BN-negative leaves of grapevine cv. Sangiovese sampled: (**A**) in July (symptoms of disease on leaves were not yet visible) and (**B**) in September (symptoms of disease on leaves were clearly visible). The statistical analysis between BN-positive and BN-negative leaves was carried out using a multiple *t*-test (FDR = 5%) and significant differences at *p* < 0.0001 are marked by four asterisks. Values are reported as means and standard deviation of five harvested samples (n = 5 BN-positive and BN-negative plants, respectively), each measured in three technical replicates. BN = Bois noir phytoplasma.

*Flavan-3-ols, flavonols and stilbenoid*. Figure 4 reports the amounts of the other secondary metabolites quantified in leaves of grapevine cv. Sangiovese sampled in the two key-stages (July and September). 

As shown in Figure 4, significant differences in the amounts of flavan-3-ols were observed between BN-positive and BN-negative leaves sampled in September. In fact, the amounts of catechin (n. **10**) and epicatechin (n. **15**) measured in July in BN-positive and BN-negative leaves were almost the same and no significant difference was observed. In September the amounts of catechin (n. **10**) and epicatechin (n. **15**) increased significantly only in BN-positive leaves, which were 1.5 and 4-fold higher than BN-negative leaves, respectively. Flavonols were the prevalent group detected in grapevine leaf extracts (Table 2) and four different quercetin-glycosides were quantified (quercetin-glucoside (n. **26, 28**), quercetin 3-glucuronide (n. **27**), quercetin 3-*O*-rhamnoside (n. **33**), and quercetin (n. **38**)). Of all the flavonols quantified, only quercetin had a significantly higher concentration (about 2-fold higher) in BN-negative leaves sampled in July than BN-positive leaves which still showed no symptoms attributable to phytoplasma disease. In September, an alteration in the amount of flavonols in the BN-positive symptomatic leaves was not observed although the symptoms of disease on the leaves were clearly visible (Figure 4). Furthermore, the content of resveratrol glucoside (n. **36**) was quantified in the BN-positive and BN-negative samples in the two key-stages of July and September, and in the presence and absence of disease symptoms on the leaves, respectively (Figure 1). 

As shown in Figure 4, the amount of resveratrol glucoside (n. **36**) in BN-positive leaves was noticeably higher in September and statistically greater than in BN-negative leaves. Specifically, in September the amount of resveratrol glucoside (n. **36**) in BN-positive leaves was more than twofold higher than the BN-negative leaves (Figure 4).

### 2.5. Lignin Distribution in BN-Positive and BN-Negative Sangiovese Leaves

**Figure 5:**
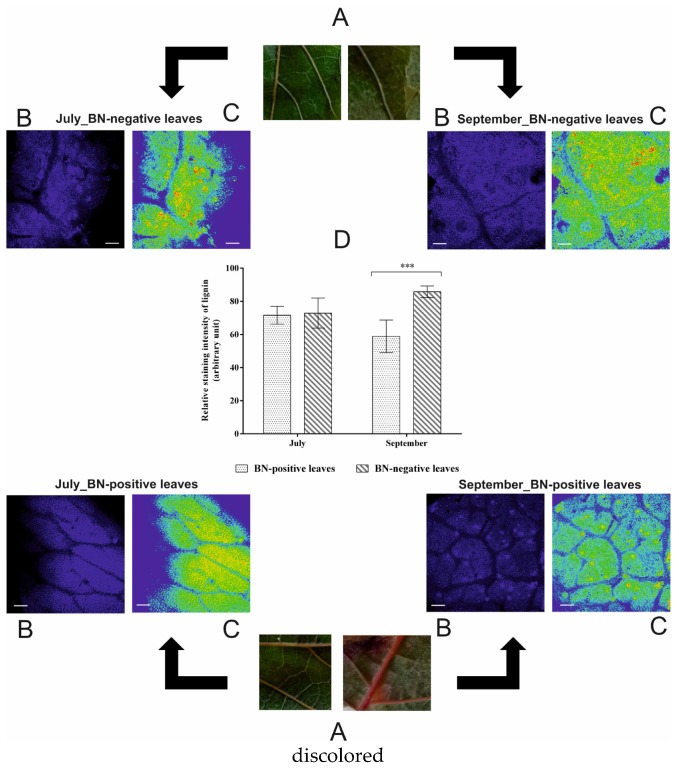
(**A**,**B**) Phenotype and lignin auto-fluorescence of hand cross sections of BN-positive and BN-negative leaves of grapevines cv. Sangiovese in July and in September; (**C**) Intensities of autofluorescence signals are represented in false rainbow colors (highest intensity in red and the lowest intensity in blue); (**D**) Quantification of the intensity of autofluorescence signal. The statistical analysis between BN-positive and BN-negative was carried out using multiple *t*-test (FDR = 5%) and significant differences at *p* < 0.001 are marked by three asterisks. Bar: 100 µm.

Histological observations of cv. Sangiovese leaves showed that the impact of the phytoplasma disease on lignin distribution differed between the July and September sampling periods (Figure 5). A statistically significant decrease in lignin (−31%) was reported only in BN-positive leaves sampled in September, while no significant difference was found in lignin distribution between BN-positive and BN-negative leaves sampled in July (Figure 5).

## 3. Discussion

Some pathogens, including phytoplasma of grapevine, can affect the carbohydrate metabolism of host plants and, consequently, induce other important metabolic alterations [12,38]. These include modifications in photosynthesis and secondary metabolism, especially a modification in the phenylpropanoid biosynthesis with a reduction and/or accumulation of some plant secondary metabolites, such as anthocyanin, phenolic compounds, and lignin [15,17,18]. The most common symptom of grapevine infected by phytoplasma is leaf yellowing (in white-berried grapes) or reddening (in red-berried grapes) caused by extensive modification in the synthesis and transport of soluble carbohydrates and starch. In fact, there is evidence of the anomalous accumulation of carbohydrates, the decrease in photosynthetic activity, the breakdown of chlorophyll, carotenoids and their biosynthesis inhibition in many phytoplasma-infected plants [8,10,11,12,13]. 

Our findings on the leaves of red-berried grape cv. Sangiovese confirmed an increase in soluble sugar content and a reduction in the chlorophyll content of the leaves positive to the pathogen, in accordance with data in the literature on white-berried grape cv. Chardonnay [11,39,40]. In particular, an increase in the soluble sugar content was observed in the BN-positive leaves sampled in two different key stages. On the other hand, a significant reduction in chlorophyll *a* content was observed even when the characteristic symptoms of phytoplasma disease were not yet visible on the leaves. However a drastic and significant reduction in the total content of chlorophylls and carotenoids was observed in September, when the plants infected by the pathogen show leaves with obvious symptoms (Table 1). 

Our results would seem to indicate that the accumulation of soluble sugars and the reduction in chlorophyll content in the BN-positive leaves interfere and change the secondary metabolism. In fact, the source-sink transition in relation to pathogen infection is commonly linked to coordinated defense responses and changes in secondary metabolite production [11,13,14,15,18,19,41,42].

In previously untested red-berried cv. Sangiovese, we also analyzed the qualitative profiles and respective amounts of phenolics, flavonoids, stilbenoids, and lignins produced by plant hosts in response to pathogen infection. The qualitative profiles of methanolic extracts of grapevine leaves showed no differences in either BN-positive or BN-negative samples, and the 36 compounds annotated and/or identified by HPLC ESI/MS-TOF have previously been found in grapevine leaf extracts, as reported in Table 2. 

On the other hand, statistically significant differences were revealed in the amount of some chemical classes and their single compounds grouped as phenolics and flavonoids, according to the health status and/or period of sampling. In fact, our results show a markedly increased phenolic and flavonoid production in asymptomatic or symptomatic BN-positive leaves compared to BN-negative leaves collected in the same period. However, the amount of proanthocyanidins increased only in asymptomatic BN-positive leaves sampled in July, as reported in Figure 2. 

Among the identified phenolics and flavonoids in the leaves of grapevine cv. Sangiovese (Table 2), the main secondary metabolites known as antioxidant compounds involved in defense reactions, have been quantified [13,18,40,43,44]. The most important alterations induced by BN disease in phenylpropanoid pathway products were observed in the amount of anthocyanin (cyanidin 3-glucoside (n. **41**)), flavan-3-ols (catechin (n. **10**) and epicatechin (n. **11**)), stilbenoid (resveratrol glucoside (n. **36**)), and lignin (Figure 3, Figure 4 and Figure 5). The amount of cyanidin 3-glucoside (n. **41**) quantified in the BN-positive leaves with clear symptoms of disease sampled in September was considerably higher (over 10-fold higher) compared to the BN-negative leaves sampled in the same phase. However, no statistical difference was observed in July between BN-positive compared to BN-negative leaves (Figure 3). 

Although an increase in total anthocyanins in leaves of Barbera red-berried grapevine infected by Flavescence dorée phytoplasma has already been observed [13], our results show for the first time changes in the anthocyanin profile and in particular, that only cyanidin 3-glucoside (n. **41**) accumulates in Sangiovese grapevine BN-positive leaves. Increases in the amounts of catechin (n. **10**), epicatechin (n. **15**) and resveratrol glucoside (n. **36**) were also observed in BN-positive leaves with clear symptoms of disease (Figure 3), in accordance with data reported by [17,19] on Chardonnay leaves and 1-year-old canes, and by [13] on Barbera leaves. However, higher amounts of quercetin (n. **38**) (Figure 4) were quantified in BN-negative leaves sampled in July. 

It has been reported [45] that constitutive higher amounts of flavonols, and in particular quercetin (n. **38**), could limit the diffusion of specific pathogens in grape leaves, and further investigations are necessary to understand whether the reduction in this compound could become an early indicator of the health status of grapevine, well before the appearance of symptoms. In fact, several global transcription profiles in Chardonnay and Manzoni Bianco grapevine infected with BN phytoplasma have revealed that the genes involved in the phenylpropanoid biosynthetic pathway are up-regulated in phytoplasma-infected leaves [11,16].

**Figure 6:**
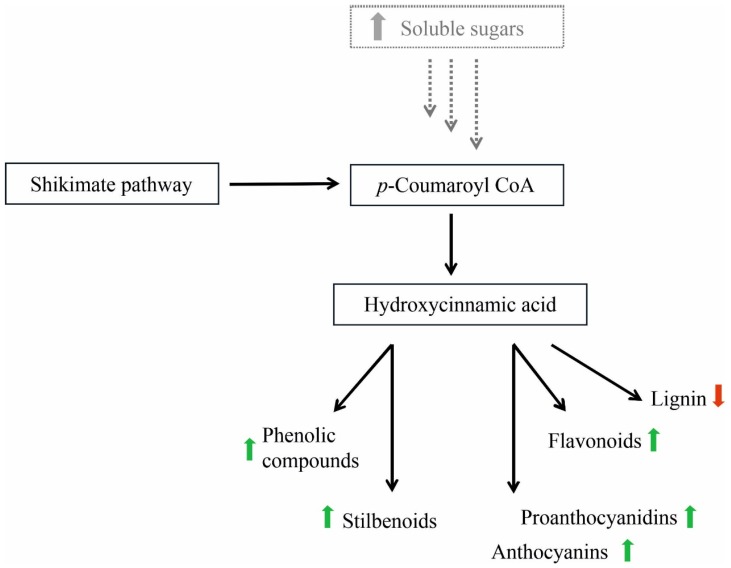
The biosynthetic pathways of phenylpropanoids and possible alterations in grapevine cv. Sangiovese leaves infected by Bois noir phytoplasma in relation to the increase in soluble sugars. Green arrows indicate the increase in metabolites, red arrows indicate the decrease in metabolites.

Further evidence of an alteration in the biosynthetic phenylpropanoid pathway products in grapevine cv. Sangiovese infected by BN, was a statistically significant difference in the content of lignin. In fact, as shown in Figure 5, in September the lignin content in BN-positive leaves was significantly lower than in BN-negative leaves. This suggests that in response to phytoplasma invasion, the synthesis of phenolic compounds, flavonols, and lignin uses the same precursor pool (hydroxycinnamic acid) and, thus, their excessive use can affect the response of other pathways, as observed in *Vitis* spp. and other plant species in relation to pathogen disease (see Figure 6) [19,46].

Although all these secondary metabolites have long been recognized as molecules implicated in the defense mechanisms activated by the host plant, they do not seem to be beneficial to the cv. Sangiovese infected by BN phytoplasma despite their biosynthesis being either activated or inhibited in BN-positive compared with BN-negative leaves. Further studies are therefore needed to understand whether or not these bio-compound accumulations have any direct protective role toward the establishment of phytoplasma infection.

## 4. Materials and Methods

### 4.1. Plant Samples and Phytoplasma Detection 

The study was conducted during 2018 within an experimental area selected as representative of a vineyard located in Tuscany (central Italy), where BN-positive and BN-negative plants were detected through multi-year monitoring (see following Section 4.2 for details on tests). Sampling was carried out at two different key-stages according to symptom appearance on leaves (July and September, respectively with asymptomatic or symptomatic leaves). Sampling was performed on the same plants all the time, collecting 10–15 leaves from five BN-positive plants and five BN-negative plants. In both sampling periods, the severity of symptoms was classified according to a grapevine yellows symptomatic scale from 0 to 3, as reported in [47]: (i) symptom severity class 0 = plants with no symptoms, (ii) symptom severity class 1 = one shoot with mild leaf symptoms, (iii) symptom severity class 2 = two to three shoots with leaf symptoms, and (iv) symptom severity class 3 = more than three shoots with symptoms of reddening leaf and berry shrivel.

The leaves collected were stored at −20 °C until DNA extraction for phytoplasma detection or lyophilized (Christ alpha 2-4 LSC plus, Osterode am Harz, Germany) for biochemical analysis. 

The DNA was extracted according to [48] with some modifications reported in [49]. Specific detection of BN phytoplasma was carried out by amplification of 16S ribosomal DNA through TaqMan assay following reaction conditions, as described in [50]. The grapevine *chloroplast chaperonin 21* gene and DNA extracted from BN-negative and BN-positive plants were used as endogenous, negative and positive controls, respectively. A threshold cycle of <37 was associated with the presence of BN phytoplasmas [50]. 

Both BN-positive and BN-negative plants were tested for some of the most common viruses of *Vitis* spp. (European Commission directive 2005/43/EC). Diagnostic tests (real-time PCR) were carried out for *Grapevine fanleaf virus* (GFLV), *Arabis mosaic virus* (ArMV), *Grapevine leafroll associated-virus 1* (GLRaV-1), *Grapevine leafroll associated-virus 3* (GLRaV-3), and *Grapevine fleck virus* (GFkV) [51,52,53]. Both BN samples (BN-negative and BN-positive) were collected from plants negative to all diagnostic tests conducted. In addition, protection of BN-positive and BN-negative plants was carried out according to common practices in the area, and sampled plants showed no symptoms related to *Uncinula necator* (Schw.) Burr., *Plasmopara viticola* (Berk. *et* Curt.) Berl. *et* de Toni, *Botrytis cinerea* Pers, and *Guignardia bidwellii* (Ellis) Viala & Ravaz.

### 4.2. Analysis of Pigments and Soluble Sugars 

Chlorophylls and carotenoids were extracted from lyophilized leaves and homogenized using liquid nitrogen with 80% acetone at a ratio of 1:100 (w/v) under stirring for 30 min. The amounts of chlorophylls and carotenoids were calculated by applying the formula in [54] and the absorbance was read with a JASCO V-550 UV/VIS spectrophotometer (JASCO Corporation 2967-5, Ishikawa-machi, Hachioji-shi Tokyo, Japan). The results were expressed in mg·g^−1^ dry weight (DW), and all measurements were performed in triplicate for each analyzed sample (n = 5, BN-positive and BN-negative plants, respectively).

The content of soluble sugars, expressed as mg·g^−1^ DW, was calculated using a commercial enzymatic kit from Megazyme (Megazyme International Ltd., Ireland, cat. no. K-SUFRG 06/14), according to the manufacturer’s protocol.

### 4.3. Total Phenolic Content (TPC), Total Flavonoid Content (TFC), and Proanthocyanidins (PAs)

The collected leaves were lyophilized and ground with a mortar and pestle in liquid nitrogen to which the extraction buffer (methanol:water:formic acid, 60:39.9:0.1) at a ratio of 1:10 was added, and left to stir in the dark for 2 h. After centrifugation at a maximum speed (5000× *g*), the resulting solutions were filtered into glass vials using a 0.2 µm polytetrafluoroethylene (PFTE) membrane and analyzed as described below. Three replicates for each harvested sample (n = 5, BN-positive, and BN-negative plants, respectively) were carried out. 

The total phenolic content (TPC) was determined using the spectrophotometric Folin–Ciocalteau method [55] and the data were expressed as mg of caffeic acid equivalent (CAE)·g^−1^ DW.

The total flavonoid content (TFC) and amount of proanthocyanidins (PAs) were evaluated as reported by [56] and the absorbance was read with a JASCO V-550 UV/VIS spectrophotometer. The TFC amount was calculated by determining the absorbance at 280 and 540 nm and reported as mg of catechin equivalent (CE)·g^−1^ DW. The proanthocyanidin (PA) quantification was measured before and after hydrolysis into cyanidins (HCl 12 N with 300 mg·L^−1^ of FeSO_4_x7H_2_O for 50 min in a thermostatic bath at 100 °C at reflux). The results were expressed as mg·g^−1^ DW.

### 4.4. HPLC ESI/MS-TOF Analysis 

The phenolic characterization and quantification on leaf extracts (see Section 2.3) were performed using an Agilent 1200 High Performance Liquid Chromatography (HPLC) System (Agilent Technologies, Palo Alto, CA, USA) equipped with a standard autosampler and an Agilent Zorbax Extend-C18 analytical column (5 × 2.1 cm, 1.8 µm), as reported by [28,36]. The HPLC system was coupled to an Agilent diode-array detector (wavelength 280 nm) and an Agilent 6320 TOF mass spectrometer equipped with a dual ESI interface (Agilent Technologies) operating in negative ion mode. Detection was carried out within a mass range of 50–1700 m/z. Accurate mass measurements of each peak from the total ion chromatograms (TICs) were obtained by means of an ISO Pump (Agilent G1310B) using a dual nebulizer ESI source that introduces a low flow (20 µL min^−1^) of a calibration solution containing the internal reference masses at m/z 112.9856, 301.9981, 601.9790, and 1033.9881, in negative ion mode. The anthocyanins were identified with the same method, but with positive ionization (detection wavelength 280 and 520 nm), using the internal reference masses at m/z 121.050873, 149.02332, 322.048121, and 922.009798, as reported by [57].

The compounds were quantified using calibration curves of authentic standards (catechin, epicatechin, quercetin, kaempferol 3-O-glucoside, resveratrol glucoside, cyanidin 3-glucoside).

### 4.5. Histology: Lignin Distribution in Leaves

Free-hand cross sections of BN-positive and BN-negative fresh leaves were mounted onto microscope slides for observation under a microscope. Lignin auto-florescence was detected using UV-excitation under DAPI-filter. Images were taken on a confocal laser-scanning microscope (Carl Zeiss LSM 700 laser scanning microscope, Jena, Germany). For each section, several images were captured in order to obtain a representative distribution of lignin throughout the leaf layer (including signals captured from leaf veins); all images were acquired with the same microscope settings and analyzed with the Zeiss LSM image examiner software v. 4.2.0.121. The intensity of autofluorescence signal was quantified using Image J software. 

### 4.6. Statistical Analysis

All data were reported as the mean ± SD in triplicate for each analyzed sample (n = 5, BN-positive and BN-negative plants, respectively). The statistical analysis was performed using multiple t-tests (FDR = 5%) to highlight the differences between BN-positive and BN-negative leaves for each physiological parameter analyzed. Statistical analyses were performed using GraphPad v. 6.01.

## 5. Conclusions

We believe that this is the first biochemical description of leaves of grapevine cv. Sangiovese infected by BN phytoplasma. Our study highlighted the modulation of the phenylpropanoid biosynthetic pathway through the accumulation and/or reduction of some end-products as a defense response status. Our findings represent a starting point for future insights regarding the role of these metabolites in phytoplasma pathogenesis and the response of grapevine cv. Sangiovese. Further studies are needed to clarify the possible benefits of the accumulation or reduction of the main metabolites in relation to phytoplasma survival and the same host plant.

## Figures and Tables

**Table 1:** Differences in the content (mg·g^−1^ dry weight (DW)) of chlorophyll (Chls), carotenoids (Cars), their ratio and soluble sugars in BN-positive and BN-negative leaves collected in July and September.

	July				September			
Parameter (mg·g^−1^ DW)	BN-*p*	BN-*n*	BN-*p* vs. BN-*n*	%var.	BN-*p*	BN-*n*	BN-*p* vs. BN-*n*	%var.
Chl *a*	1.29 ± 0.01	1.52 ± 0.04	****	−15	0.57 ± 0.06	1.01 ± 0.03	****	−44
Chl *b*	0.66 ± 0.04	0.67 ± 0.05	ns	−1	0.46 ± 0.06	0.66 ± 0.03	***	−30
Chls *a*+*b*	1.95 ± 0.17	2.19 ± 0.37	ns	−11	1.03 ± 0.21	1.67 ± 0.35	**	−38
Chls *a*/*b*	1.96 ± 0.01	2.29 ± 0.01	-	−14	1.23 ± 0.01	1.53 ± 0.01	-	−19
Cars	0.25 ± 0.06	0.25 ± 0.05	ns	0	0.12 ± 0.02	0.18 ± 0.05	*	−33
Cars/Chls	0.13 ± 0.01	0.11 ± 0.02	-	-	0.12 ± 0.01	0.11 ± 0.02	-	-
Soluble sugars	80.4 ± 6.02	47.9 ± 3.21	****	+68	68.3 ± 5.93	52.2 ± 3.39	***	+31

Note: Data are presented as means and standard deviations of five harvested samples (n = 5 BN-positive and BN-negative plants, respectively) each measured in three technical replicates. The values shown in the %var columns represent the percentage reduction or increase in BN-positive sample compared to BN-negative control. The statistical analysis (BN-*n* vs. BN-*p*) was carried out using a multiple *t*-test (False Discovery Rate, FDR = 5%). BN-*p* = Bois noir positive leaves; BN-*n* = Bois noir negative leaves. Statistical significances are reported: * *p* < 0.05, ** *p* < 0.01, *** *p* < 0.001, **** *p* < 0.0001. ns = no significance.

**Table 2:** List of putative compounds and anthocyanins extracted from *Vitis vinifera* cv. Sangiovese BN-positive and BN-negative leaves detected by HPLC ESI/MS-TOF. BN = Bois noir phytoplasma.

N.	Compound (Negative Ion Mode)	RT ^a^ (min)	*m/z* exp. ^b^	*m/z* calc. ^c^	(M-H)^-^	Error ^d^	Score ^e^	Reference
1	protocatechuic acid 3-glucoside	2.451	315.0737	315.0722	C_13_H_15_O_9_	−4.98	96.36	[24,25]
2	caftaric acid isomer 1	3.060	311.0436	311.0409	C_13_H_11_O_9_	−8.76	94.65	[24,26,27]
3	caftaric acid isomer 2	3.386	311.0404	311.0409	C_13_H_11_O_9_	1.49	94.65	[24,26,27]
4	protocatechuic acid	3.689	153.0571	153.0557	C_8_H_9_O_3_	−9.28	98.83	[25,27,28]
5	protocatechuic acid glucoside	3.704	315.1101	315.1045	C_14_H_19_O_8_	−5.05	95.00	[25,26]
6	rosmarinic acid	4.610	359.0785	359.0772	C_18_H_15_O_8_	−3.46	94.79	[29]
7	caffeic acid glucoside	4.689	341.0893	341.0878	C_15_H_17_O_9_	−4.45	87.82	[25,30]
8	tartaric acid	5.176	149.0103	149.0092	C_4_H_5_O_6_	−7.56	99.85	[31]
9	coumaric acid	5.245	163.0398	163.0401	C_9_H_7_O_3_	1.87	98.83	[24,27]
10	catechin*	5.307	289.0695	289.0718	C_15_H_13_O_6_	7.67	98.49	[24,26,27]
11	isorhamnetin 3-glucuronide isomer 1	5.568	491.0841	491.0831	C_22_H_19_O_1_	−2.09	94.41	[32]
12	viniferin	6.555	453.1348	453.1337	C_28_H_21_O_6_	2.42	88.00	[33]
13	unknown	6.907	447.1492	447.1508	C_19_H_27_O_1_	3.57	43.82	-
14	unknown	7.107	451.2193	451.2185	C_20_H_35_O_1_	−1.90	90.34	-
15	epicatechin*	7.193	289.0735	289.0718	C_15_H_13_O_6_	−6.02	91.43	[24,27]
16	myricetin 3-*O*-glucuronide is.1	7.333	493.0601	493.0624	C_21_H_17_O_1_	4.67	88.49	[26]
17	hexose derivative isomer1	7.361	431.1920	431.1923	C_20_H_31_O_1_	0.65	92.50	[34]
18	hexose derivative isomer 2	7.704	431.1928	431.1923	C_20_H_31_O_1_	−1.15	99.00	[34]
19	quercetin-pentoside	7.872	433.2081	433.2079	C_20_H_33_O_1_	−0.31	94.94	[24,26,35]
20	hexose derivative isomer 3	8.073	431.1914	431.1923	C_20_H_31_O_1_	−1.98	95.00	[34]
21	unknown	8.243	657.1084	657.1097	C_30_H_25_O_1_	1.94	94.94	-
22	myricetin 3-*O*-glucuronide isomer 2	8.257	493.0629	493.0624	C_21_H_17_O_1_	−1.05	86.75	[26]
23	myricetin 3-*O*-glucuronide isomer 3	8.505	493.0643	493.0624	C_21_H_17_O_1_	−3.86	91.97	[26]
24	myricetin-3-glucoside	8.604	479.0849	479.0831	C_21_H_19_O_1_	−3.73	91.89	[27]
25	unknow	8.967	387.2021	387.2024	C_19_H_31_O_8_	0.86	92.39	-
26	quercetin-glucoside isomer 1*	9.488	463.0902	463.0882	C_21_H_19_O_1_	−4.43	93.41	[24,26,30]
27	quercetin 3-glucuronide	9.615	477.0697	477.0675	C_21_H_17_O_1_	−4.77	95.94	[26,27]
28	quercetin-glucoside isomer 2*	9.750	463.0900	463.0882	C_21_H_19_O_1_	−3.91	93.21	[24,26,30]
29	kaempferol 3-*O*-glucoside*	10.274	447.0955	447.0933	C_21_H_19_O_1_	−5.00	91.62	[24,26]
30	unknown	10.435	549.2544	549.2553	C_25_H_41_O_1_	1.62	62.74	-
31	kaempferol-rutinoside	10.519	593.1513	593.1512	C_27_H_29_O_1_	−0.10	90.62	[24,30]
32	kaempferol 3-*O*-glucuronide	10.673	461.0740	461.0725	C_21_H_17_O_1_	−3.23	91.22	[26]
33	quercetin 3-*O*-rhamnoside	10.678	447.0960	447.0933	C_21_H_19_O_1_	−6.05	93.66	[30,34]
34	unknown	10.734	429.1778	429.1776	C_20_H_29_O_1_	−2.71	88.77	-
35	isorhamnetin 3 glucuronide isomer 2	11.007	491.0841	491.0831	C_22_H_19_O_1_	−2.01	92.57	[32]
36	resveratrol glucoside	11.122	389.1228	389.1242	C_20_H_21_O_8_	3.49	95.85	[30]
37	caffeic acid and catechin condensation product	11.622	451.1010	451.1028	C_24_H_19_O_6_	−3.99	87.00	[33]
38	quercetin	13.100	301.0939	301.0412	C_15_H_9_O_7_	6.44	91.77	[36]
39	syringetin-3-*O*-galactoside	13.990	507.2089	507.2083	C_23_H_23_O_1_	−1.11	96.44	[37]
**N.**	**Compound** **(Positive Ion Mode)**	**RT ^a^ (min)**	***m/z* exp. ^b^**	***m/z* calc. ^c^**	**(M-H)^+^**	**Error ^d^**	**Score ^e^**	**Reference**
40	delphinidin 3-glucoside	8.330	465.1032	465.1028	C_21_H_21_O_1_	−0.94	98.36	[26,35]
41	cyanidin 3-glucoside*	9.615	449.1104	449.1078	C_21_H_21_O_1_	−5.78	96.25	[35]
42	peonidin 3-glucoside	12.200	463.1232	463.1235	C_22_H_23_O_1_	0.30	97.02	[35]

**^a^** Retention time, **^b^** m/z experimental, **^c^** m/z calculated, **^d^** Difference between the observed mass and the theoretical mass of the compound (ppm), **^e^** Isotopic abundance distribution match: a measure of the probability that the distribution of isotope abundance ratios calculated for the formula matches the measured data. *Confirmed by authentic chemical standards.
